# Supplementary material for: A Food Insecurity Systematic Review: Experience from Malaysia
Source: Nutrients. 2021 Mar 15;13(3):945. doi: 10.3390/nu13030945 (PMC7998204; doi:10.3390/nu13030945)
Supplement: Supplementary file 1 [file nutrients-13-00945-s001.pdf]

Table S1. Studies that met the criteria of checklist for analytic cross-sectional study.

No	Checklist	Study (Number of studies)
	Checklist for analytic cross-sectional study	
1.	Were the criteria for inclusion in the sample clearly defined?	Chan, Faller, Lau, & Gabriel, 2020 [86]; Chong, Geeta, Norhasmah, 2018 [87]; Chong, Geeta, Norhasmah, 2019 [88]; Fadilah, Norhasmah, Zalilah, & Zuriati, 2017 [89]; Ihab et al., 2012a [54]; Ihab et al., 2012c [90]; Ihab et al., 2013 [91]; Izwan Syafiq, Asma, Nurzalinda, Rahijan, & Siti Nur Afifah, 2019 [92]; Khairil, Noralanshah, Farah Syafeera, Nazrul, & Muhammad Ghazali, 2015 [93]; Mohamad Hasnan et al., 2020 [94]; Mohamadpour, Mohd Shariff, Avakh Keysami, 2012 [95]; Nor Syaza Sofiah & Norhasmah, 2020 [96]; Noratikah, Norhasmah, & Siti Farhana, 2019 [97]; Norhasmah, Zalilah, & Rohana, 2012a [98]; Norhasmah, Zuroni, & Siti Marhana, 2013 [99]; Norhasmah et al., 2011 [100]; Nurfahilin, & Norhasmah, 2015 [101]; Nur Atiqah, Norazmir, Khairil Anuar, Mohd Fahmi, & Norazlanshah, 2015 [102]; Nurulhudha, Norhasmah, Siti Nur'Asyura, & Shamsul Azahari, 2020 [103]; Nurzetty Sofia et al., 2017 [104]; Rohida, Suzana, Norhayati, & Hanis Mastura, 2017 [105]; Roslee, Lee, Nurul Izzati, & Siti Masitah, 2019 [106]; Ruhaya et al. 2020 [107]; Siti Farhana, Norhasmah, Zalilah, & Zuriati, 2018 [108]; Siti Farhana, Norhasmah, Zalilah, & Zuriati, 2020 [109]; Susanti, Norhasmah, Fadilah, & Siti Farhana, 2019 [110]; Zalilah & Ang, 2001 [111]; Zalilah & Khor, 2004 [112]; Zalilah & Khor, 2005 [113]; Zalilah & Khor, 2008 [114]; Zalilah et al., 2014 [115] (31 articles)
2.	Were the study subjects and the setting described in detail?	Chong et al., 2018 [88]; Chong et al., 2019 [89]; Cooper, 2013 [116]; Fadilah et al., 2017 [89]; Ihab et al., 2012b [117]; Ihab et al., 2012c [90]; Ihab et al., 2013 [91]; Nik Aida Adibah & Norhasmah, 2013 [118]; Nor Syaza Sofiah & Norhamah, 2020 [96]; Noratikah et al., 2019 [97]; Norhasmah, Zalilah, Kandiah, Mohd Nasir, & Asnarulkhadi, 2012b [119]; Norhasmah et al., 2011 [100]; Nurfahilin, & Norhasmah, 2015 [101]; Nurulhudha et al., 2020 [103]; Rohida et al., 2017 [105]; Roselawati, Wan Azdie, Aflah, Jamalludin, & Zalilah, 2017 [120]; Ruhaya et al. 2020 [107]; Siti Farhana et al., 2018 [108]; Siti Marhana & Norhasmah, 2012 [121]; Siti Marhana, Norhasmah, & Hsuniyah, 2014 [122]; Susanti et al., 2019 [110]; Wan Azdie, Shahidah, Suriati, & Rozlin, 2019 [123]; Wan Azdie, Shahidah, Suriati, & Rozlin, 2019 [124]; Yong & Norhasmah, 2016 [125]; Zalilah & Ang, 2001 [111]; Zalilah & Khor, 2004 [112]; Zalilah & Khor, 2005 [113]; Zalilah & Khor, 2008 [114]; Zalilah & Tham, 2002 [126]; Zalilah et al., 2014 [115] (29 articles)
3.	Was the exposure measured in a valid and reliable way?	Chan et al, 2020 [86]; Chong et al., 2018 [87]; Chong et al., 2019 [88]; Ihab et al., 2012a [54]; Ihab et al., 2012b [117]; Ihab et al., 2012c [90]; Ihab et al., 2013 [91]; Khairil et al., 2015 [93]; Mohamad Hasnan et al., 2020 [94]; Mohamadpour et al., 2012 [95]; Nik Aida Adibah & Norhasmah, 2013 [118]; Nor Syaza Sofiah & Norhasmah, 2020 [96]; Noratikah et al., 2019 [97]; Norhasmah et

		al., 2012a [98]; Norhasmah et al., 2012b [119]; Norhasmah et al., 2011 [100]; Nurfahilin, & Norhasmah, 2015 [101]; Nurulhudha et al., 2020 [103]; Nurzetty Sofia et al., 2017 [104]; Roselawati et al, 2017 [120]; Siti Marhana & Norhasmah, 2012 [121]; Ruhaya et al. 2020 [107]; Siti Farhana et al., 2020 [109]; Susanti et al., 2019 [110]; Yong & Norhasmah, 2016 [125]; Zalilah & Ang, 2001 [111]; Zalilah & Khor, 2004 [112]; Zalilah & Khor, 2005 [113]; Zalilah & Khor, 2008 [114]; Zalilah & Tham, 2002 [126]; Zalilah et al., 2014 [115] (31 article)
4.	Were objective, standard criteria used for measurement of the condition?	Alam, Siwar, Wahid, & Abdul Talib, 2015 [127]; Chong et al., 2018 [87]; Chong et al., 2019 [88]; Cooper, 2013 [116]; Fadilah et al., 2017 [89]; Ihab et al., 2012a [54]; Ihab et al., 2012b [117]; Ihab et al., 2012c [90]; Ihab et al., 2013 [91]; Nik Aida Adibah & Norhasmah, 2013 [118]; Noratikah et al., 2019 [97]; Norhasmah et al., 2012a [98]; Norhasmah et al., 2012b [119]; Nur Atiqah et al, 2015 [102]; Nurfahilin, & Norhasmah, 2015 [101]; Nurulhudha et al., 2020 [103]; Rohida et al., 2017 [105]; Roselawati et al., 2017 [120]; Ruhaya et al. 2020 [107]; Siti Farhana et al., 2018 [108]; Siti Farhana et al., 2020 [109]; Siti Marhana & Norhasmah, 2012 [121]; Siti Marhana et al., 2014 [122]; Susanti et al., 2019 [110]; Yong & Norhasmah, 2016 [125]; Zalilah & Ang, 2001 [111]; Zalilah & Khor, 2008 [114]; Zalilah et al., 2014 [115] (28 articles)
5.	Were confounding factors identified?	Chong et al., 2018 [87]; Chong et al., 2019 [88]; Ihab et al., 2012a [54]; Ihab et al., 2012b [117]; Ihab et al., 2012c [90]; Ihab et al., 2013 [91]; Mohamadpour et al., 2012 [95]; Noratikah et al., 2019 [97]; Norhasmah et al., 2011 [100]; Norhasmah et al., 2012a [98]; Nur Atiqah et al, 2015 [102]; Nurulhudha et al., 2020 [103]; Nurzetty Sofia et al., 2017 [104]; Roselawati et al., 2017 [120]; Ruhaya et al. 2020 [107]; Siti Farhana et al., 2020 [109]; Susanti et al., 2019 [110]; Zalilah & Khor, 2004 [112]; Zalilah & Khor, 2005 [113]; Zalilah & Khor, 2008 [114]; Zalilah et al., 2014 [115] (21 articles)
6.	Were strategies to deal with confounding factors stated?	Chong et al., 2018 [87]; Chong et al., 2019 [88]; Ihab et al., 2012a [54]; Ihab et al., 2012b [117]; Ihab et al., 2012c [90]; Ihab et al., 2013 [91]; Mohamadpour et al., 2012 [95]; Noratikah et al., 2019 [97]; Norhasmah et al., 2011 [100]; Norhasmah et al., 2012a [97]; Nur Atiqah et al, 2015 [102]; Nurulhudha et al., 2020 [103]; Nurzetty Sofia et al., 2017 [104]; Roselawati et al., 2017 [120]; Ruhaya et al. 2020 [107]; Siti Farhana et al., 2020 [109]; Susanti et al., 2019 [110]; Zalilah & Khor, 2004 [112]; Zalilah & Khor, 2005 [113]; Zalilah & Khor, 2008 [114]; Zalilah et al., 2014 [115] (21 articles)

7.	Were the outcomes measured in a valid and reliable way?	Chan et al, 2020 [86]; Chong et al., 2019 [88]; Mohamad Hasnan et al., 2020 [94]; Nor Syaza Sofiah & Norhasmah, 2020 [119]; Noratikah et al., 2019 [120]; Nur Atiqah et al, 2015 [95]; Roslee et al., 2019 [102]; Susanti et al., 2019 [128]; Zalilah & Ang, 2001 [111]; Zalilah & Khor, 2004 [112] (10 articles)
8.	Was appropriate statistical analysis used?	Chan et al, 2020 [86]; Chong et al., 2018 [87]; Chong et al., 2019 [88]; Fadilah et al., 2017 [89]; Ihab et al., 2012a [54]; Ihab et al., 2012b [117]; Ihab et al., 2012c [90]; Ihab et al., 2013 [91]; Khairil et al., 2015 [93]; Mohamad Hasnan et al., 2020 [94]; Mohamadpour et al., 2012 [95]; Nor Syaza Sofiah & Norhasmah, 2020 [96]; Noratikah et al., 2019 [97]; Norhasmah et al., 2011 [100]; Norhasmah et al., 2012a [98]; Nurulhudha et al., 2020 [103]; Nurzetty Sofia et al., 2017 [104]; Roselawati et al., 2017 [120]; Roslee et al., 2019 [106]; Ruhaya et al. 2020 [107]; Siti Farhana et al., 2018 [108]; Siti Farhana et al., 2020 [109]; Susanti et al., 2019 [110]; Zalilah & Khor, 2004 [112]; Zalilah & Khor, 2005 [113]; Zalilah & Khor, 2008 [114]; Zalilah et al., 2014 [115] (27 articles)

Table S2. Studies that met the criteria of checklist for qualitative research.

No	Checklist	Study (Number of studies)
	Checklist for qualitative research	
1.	Is there congruity between the stated philosophical perspective and the research methodology?	Law, Norhasmah, Gan, & Mohd Nasir, 2018a [127] (1 article)
2.	Is there congruity between the research methodology and the research question or objectives?	Law, Roselan, & Norhasmah, 2015 [128]; Law et al., 2018a [127]; Law, Norhasmah, Gan, Siti Nur'Asyura, & Mohd Nasir, 2018b [129]; Norhasmah, Zalilah, Mohd Nasir, Asnarulkhadi, 2010 [130] (4 articles)
3.	Is there congruity between the research methodology and the methods used to collect data?	Law et al., 2015 [128]; Law et al., 2018a [127]; Law et al., 2018b [129]; Norhasmah et al., 2010 [130] (4 articles)
4.	Is there congruity between the research methodology and the representation and analysis of data?	Law et al., 2015 [128]; Law et al., 2018a [127]; Law et al., 2018b [129]; Norhasmah et al., 2010 [130] (4 articles)
5.	Is there congruity between the research methodology and the interpretation of results?	Law et al., 2015 [128]; Law et al., 2018a [127]; Law et al., 2018b [129]; Norhasmah et al., 2010 [130] (4 articles)

6.	Is there a statement locating the researcher culturally or theoretically?	(0 articles)
7.	Is the influence of the researcher on the research, and vice- versa, addressed?	Law et al., 2018b [129]<br>(1 article)
8.	Are participants, and their voices, adequately represented?	Law et al., 2018a [127]; Law et al., 2018b [129]; Norhasmah et al., 2010 [130]<br>(3 articles)
9.	Is the research ethical according to current criteria or, for recent studies, and is there evidence of ethical approval by an appropriate body?	Law et al., 2018a [127]; Law et al., 2018b [129]; Norhasmah et al., 2010 [130]<br>(3 articles)
10.	Do the conclusions drawn in the research report flow from the analysis, or interpretation, of the data?	Law et al., 2015 [128]; Law et al., 2018a [127]; Law et al., 2018b [129]; Norhasmah et al., 2010 [130]<br>(4 articles)
